# The Mineral Nutrients, Air, Water and the Salts1Read before the Medical Club, March 18, 1908.

**Published:** 1909-01

**Authors:** Henry Reed Hopkins

**Affiliations:** Buffalo, N. Y.; Ninety-Seventh President of the Medical Society of the State of New York


### BUFFALO MEDICAL JOURNAL.

VOL. LXIV. JANUARY, 1909. No. 6

### ORIGINAL COMMUNICATIONS.

### The Mineral Nutrients, Air, Water and the Salts.[1]

BY HENRY REED HOPKINS, M. D., Buffalo, N. Y.

Ninety-Seventh President of the Medical Society of the State of New York.

THE question of our daily bread is one of elemental importance, to the sick and to the well, in all classes and in all ages, for prevention and for cure. Its consideration may properly begin with a word of caution against the work of the optimist, the pessimist, the medical anarchist, and more than all others, because more numerous than all the rest, the over credulous, the brain weary, the pseudo-scientists, the Quimby-Eddyites. All of these are at once the enemy to be conquered, the heathen to be converted, the children to be won and protected, by sound teaching and right example upon our part.

In addition to its primary importance, the knowledge of dietetics is interesting from the fact that it is sought and used by all classes of doctors,—by physicians, by homeopaths, by eclectics, and by the osteopaths. The Quimby-Eddyites are the only medical cult of any considerable number that openly flaunt the importance of right ideas and practises in dietetics; on the other hand their predecessors and introducers, the homeopaths have won the most substantial of their confidence by strict attention to these matters. Furthermore, I think it is expedient at this time that we give this topic consideration for the reason that recent advances in physico-chemistry enable students to say in truth as never before that there is a science of dietetics, and the art of dietetics should ever be willing to receive with joy whatever of practical contributions science has to offer. And, finally, to conclude this introduction, the writer has something to say which he not only believes worthy of consideration and, moreover, for that something which he now offers he earnestly desires the characteristic scrutiny and criticism from the members present without favor, and without mutual admiration, to the end that only the fit shall survive.

---

1. Read before the Medical Club, March 18, 1908.

For many years I have been troubled with the growing conviction that the role of the inorganic nutrients in animal metabolism is not adequately appreciated, at least as indicated by the teachings, the writings and the practice coming under my observation. I do not know of a writer on hygiene or dietetics who states this matter plainly and comprehensively with any approach to suitable emphasis, and of this part of medical literature generally it may be said, that it is feeble, confusing, uncertain, inadequate, incompetent. This criticism is competent only upon the assumption, the result of the best study and thought I have been able to give the matter, that the inorganic foods, the mineral nutrients, include air, water and the mineral salts found in the excretions and the bodies of animals. Also, the further assumption that when foods are classified and enumerated in the order of importance, the inorganic foods, the mineral nutrients, should stand in class one. That, as in the nutrition of plants the inorganic foods, the mineral nutrients are the foods and there are no others; even so in the metabolism of animals these same foods are far and away more essential and more important than any others; that in classification these should be placed first, and in description, these should be treated at length with the emphasis due their primary and singular importance. Perhaps it should be further observed here, that in thinking of classification of foods I am using the word in an inclusive and comprehensive sense, having in mind something of the relative importance of foods, of their intimate relation to life and to its manifestations, as sensation, growth, and repair, rather than having in mind the more accidental appearances of foods, whether they are solid or fluid, bulky or concentrated, or the like. I am thinking of classification which is inclusive and which is at least an effort to recognise in some reasonable degree the present state of knowledge as to the physico-chemistry of biology.

Under the classification of foods by various authors: Parke's Hygiene gives: (1) Nitrogenous; (2) Fats and oils; (3) Sugars; (4) Water and salts, magnesium, calcium, potassium, sodium and iron as chlorides, phosphates and sulphates.

Currier Practical Hygiene gives: (1) Water; (2) Salts; (3) Albumins; (4) Fats and oils; (5) Starch and sugars.

Mrs. Hart in Diet in Sickness and Health says: Foods are divided into two classes: (1) Nitrogenous; (2) Non-nitrogenous, and being a woman adds a postscript: Inorganic water and salts.

Coplin and Bevan, in Practical Hygiene give: (1) Nitrogenous; (2) Fats and oils; (3) Starches and sugars; (4) Inorganic compounds, and adds to the latter that these demand no comments.

Richards and Woodman, in Air, Water and Food, give: (1) Nitrogenous; (2) Fats and oils; (3) Starches and sugars; (4) Mineral salts.

Egbert, Hygiene and Sanitation, gives: (1) Proteids and albuminoids; (2) Carbohydrates; (3) Fats and oils; (4) Mineral salts.

Bergey, in Principles of Hygiene, makes no classifications of foods but recites constituents of the body inorganic and organic and then says: "All these various elements and chemical combinations constituting the composition of the body, must be supplied in the food supply in order that it may perform its normal functions and obtain energy for all of man's activities in life."

Stockton, in Personal Hygiene (Pyle), gives: (1) Nitrogenous substances: (2) Fats; (3) Carbohydrates; (4) Salts.

Rohe in Textbook of Hygiene, gives: (1) Water; (2) Salts; (3) Proteids; (4) Fats or carbohydrates.

Wilson, in Handbook of Hygiene and Sanitary Science, gives: (1) Nitrogenous albuminates; (2) Fats or oils; (3) Sugars and starches; (4) Water and saline matters.

Harrington, in Practical Hygiene, gives: (1) Proteids; (2) Fats; (3) Carbohydrates and organic acids; (4) Water and mineral salts.

### WHY AIR AND WATER ARE FOODS.

The facts which to my mind should compel us to include air and water in our list of foods, and should also give them a place at the head of that list, are found in a consideration of what are foods, what is a food? Let us listen to some definitions:

Friedenwald and Ruhräh says: "Food is matter that is taken into the body to supply nourishment, or to replace tissue waste." Bergey quotes Dr. Atwater's definition of food as follows: "Food is that which when taken into the body, builds up its tissues and keeps them in repair, or which is consumed in the body to yield energy in the form of heat to keep it warm and create strength for its work."

Hutchinson says "A food may be defined as anything which, when taken into the body, is capable of either repairing its waste or furnishing it with material from which to produce heat or nervous and muscular work." Harrington says: "Foods may be said to include everything taken into the system capable of being utilised directly or indirectly to build up normal structure, repair waste, or produce energy in any form."

Let it be noted that the descriptive terms of these definitions are distinctly inclusive in character. "That which;" anything which;" "everything taken into the system."

Again, it may be observed that the eminent writers are having in mind the essential fact that these food substances are taken into the system, and they treat as immaterial and non-essential the route to that system, be that route by nose and lungs, or be it by mouth and stomach. The destination, the system is the thing,—the route is not material.

Again, observe the inclusiveness of the phrase "capable of being utilised directly or indirectly." In these words the writer seems to have in mind a number of substances and chiefly some of the mineral salts which are incapable of oxidation, which pass into and through the system unchanged, and therefore a superficial examination might lead one to the conclusion that they could in no sense be considered foods; but upon more intimate study, it is learned that these substances are habitually found in living cells, that these substances are constantly given off from living cells, and that these substances must be constantly furnished as part of the food supply of living cells, and that therefore these substances should be included and rated as foods, hence the inclusive description "capable of being utilised directly or indirectly." It would seem that several tests or measurements are to be given any substance claiming to be a food. First, it must be some form of matter; second, it must enter the system; third, it must contribute directly or indirectly to the growth of cells or of the individual; fourth, it must aid directly or indirectly in the repair of waste; fifth, it must contribute directly or indirectly to the production of some form of vital energy.

With these tests or measurements in our minds, let us examine our substances,—air and water,—and learn if their relations to metabolism are such as fit them for places in the list of foods. We will agree that air is matter, and that it is taken into the interior of the body through a complicated and specially devised mechanism whereby one of the constituents of the air is prepared for absorption into the system, and we will further agree that a certain proportion of each separate meal of air so taken into the body, so prepared for absorption, is absorbed and the residue is promptly extruded from the body after the manner of the excrements in general. We will probably further agree that the more important constituent of air,—oxygen,—which is absorbed directly into the system is utilised directly or indirectly to build up normal structure, repair waste, and to produce energy in every form. It possibly may help some of us to make this realisation more actual, more vivid, to recall that Thompson quotes Moss as authority for the statement, that the body of a man weighing 148 pounds contains 92.4 pounds of oxygen, about three times as much as the next in weight, the carbon which weighs 31.6 pounds.

When we further recall that oxygen is an essential constituent of every tissue, fluid and cell of the body, that it is found in all of the excretions, that it is the active and energising agent in all of the chemico-metabolic reactions wherein and whereby vital energy is manifested, that when the supply of atmospheric oxygen is stopped for one minute, there is distress, for two minutes there is mortal anguish, and for three minutes there is the death of the individual, it would seem that we must conclude, not only is air food but also that air is the food.

The proof as to the right of water to be considered as an important food follows about the same lines as in the case of air, and in certain respects is more readily convincing. Many of us are dependent upon sight and smell and taste and touch in our recognition of matter, and although we may profess differently, the fact remains that it is easier for us to realise the materiality of water than it is of air. The contents of the bathtub, of the water jug, of the snow bank or of the swiftly flying snowball, or of the ponderous iceberg do not permit one to doubt that water is matter. I think all will admit that it would be quite a bit more difficult for us to reach such a conclusion if we knew of water only in its physical state of a pure gas, as found constantly present in the air. I refer to this for the reason that it seems to me quite probable that the failure of our masters and teachers to consider air as one of the foods comes in part from the unconscious reluctance to consider air as matter. Be this as it may the evidence in the case of water is easily convincing.

Neither do we need argument to convince us that water habitually enters the mouth and the system. The pleasures of the palate and that delightful something which comes to some of us after a few glasses of beer or of wine or of hot Scotch, remind us that water does enter the system and that frequently. The role of water as a factor in living matter is enormous but may be quickly stated. Water is the most abundant constituent of living matter, comprising all the way from 2 to 3 per cent. of the most dry of the tissues up to 95 per cent. or even higher of the blood and the lymph. Water serves a most important role in metabolism and growth, as an organising fluid for the colloidal matter, and as a solvent for the crystaloids, making a medium of exchange between the cells, the different tissues and organs, and the fluids of the body, serving most important uses in all metabolic processes. Water also aids directly as a nutritive substance, yielding its constituent elements for the construction of essential compounds of living matter. It further makes anabolism and katabolism possible by its action as the universal solvent, whereby all other foods are prepared for absorption for the penetration of cell membranes or walls in anabolism; and whereby all excretory substances are enabled to pass through those cell membranes or walls in katabolism. In fact, upon the peculiar properties and constant presence of water depend all of those metabolic growths, repairs, activities and energies we know as life.

Thompson gives us a most interesting table from Moss:

The chemical analysis of a man—weight, 148 pounds, and names the several weights of oxygen, 92.4 lbs., hydrogen, 14.6, carbon, 31.6, nitrogen, 4.6, phosphorus, 1.4, calcium, 2.8, sulphur, .24, chlorine, .12, sodium. .12, iron, .02, potassium, .34, magnesium, .04, and then adds this sentence which is characteristic of the best literature of the subject: "All these elements are necessarily derived from food and water plus the oxygen of the air which is breathed." In my opinion the facts of biologic chemistry not in dispute by sane people require that this statement be made more simple, more direct, more accurate after this manner.

All these elements are necessarily derived from food.

Before dismissing our consideration of the evidence as to the position of water near the head of the list of foods, it may be well to recall two quite significant facts.

1. The frequency with which water is taken by all sorts of men, as drink, as beverage, as fruit and vegetables, and as an important part of most of our so-called dry foods.

2. The most significant fact of the shortness of the time one can live without water,—five days,—as compared to the length of days,—more than 40,—one lives without the so-called foods if water is drunk![1]

THE CASE OF THE FOOD SALTS.

In addition to the four great elements,—oxygen, carbon, hydrogen, nitrogen,—there are found in living tissues calcium, potassium, sodium, magnesium and iron. These elements are found in combinations as phosphates, sulphates, carbonates and chlorides. Of these inorganic substances it is to be noted that each one is essential and indispensable to animal life, as with the exception of sodium they are essential and indispensable to plant life. Observation has demonstrated that these substances are present in many of the articles upon which men depend for food, as mineral waters, in the cereals, in vegetables and in fruits, and in all of the meats and other foods of animal origin.

Observation has also established that these substances are habitually found in man's excretions, showing thereby that man

1. Proportions of the several chemical constituents of man's body—weight 148 pounds—after Thompson and Moss: Oxygen, 4,620; to iron, 1. Carbon, 1,630; to Iron, 1. Hydrogen, 730; to Iron, 1. Nitrogen, 230; to iron, 1. Calcium, 140; to Iron 1. Phosphorus, 70; to Iron, 1. Potassium, 17; to Iron, 1. Sulphur, 12; to Iron, 1. Sodium, 6; to Iron, 1. Chlorine, 6; to Iron, 1. Magnesium 2; to Iron, 1.

needs them in his food: and, finally, experiments have demonstrated in plants and in animals alike that growth, repair of waste and metabolic functions, life is impossible without the constantly renewed presence and physico-chemical activities of these substances.

Furthermore, it is known that in plants and animals there is the faculty of nutritional forethought, the expectation that there may come a time of want, the knowledge that in times of prosperity preparation should be made against this need, and apparently for this purpose the tissues of plants and animals are capable of storing these invaluable inorganic treasures much as bees store honey and the camel stores water against a time of want. Therefore, plants and animals in case of starvation from these substances live for a time upon their accumulated stores, but these once exhausted, death is inevitable and we will do well to try and remember that death is inevitably the result of starvation from any one of this group of inorganic nutrients.

An interesting fact should be cited here in connection with Liebig's Law of the Minimum, which is to the effect that individuals, plants and animals require a certain amount of each of these mineral nutrients for growth or life, that plants and animals are capable of absorbing and usually do absorb more of each of these substances than this minimum, that these substances are capable in a slight degree of replacing each other or some of them in metabolism. For instance, with an excess of sodium an individual can live with less than the minimum of potassium, and in like manner magnesium can in a degree replace calcium, and that growth and normal function is impossible when the supply of each substance is reduced to the minimum. I have the conviction that the consideration of the metabolic relations of the mineral nutrients leads us into the arcana, the holy of holies of the art and science of nutrition, to the intimate facts of life; also that recent advances of our knowledge in this direction are illuminating, hopeful and inspiring to a degree and I would there was time that we might give consideration to some kindred and most interesting topics.

The role of the mineral nutrients in dissociation in ionisation, the importance and actions in metabolism of ions, anions and cations between cells and intercellular lymph; the relation of the mineral nutrients to surface tension, to solution tension, to permeability, to osmotic pressure, to osmosis, to the physics of absorption, to nutrition, to life, these are chapters of a paper which if well written would be worth hearing. However, we must come back and sum up the evidence, which seems to amount to proof, that the inorganic salts before named are entitled to be classed as foods, entitled to give the name to our first and most important group of foods,—the mineral nutrients,—air, water and the salts.

First, these are material substances—matter. Second, they enter the system in a wide variety of foods, mineral, vegetable and animal. Third, they are essential directly or indirectly to growth, repair of waste and the production of energy to metabolism,—to life.

It would seem that we have proved our case, that there is a class of foods which may properly be called mineral nutrients; that this class includes air, water and the salts; that this group is an important group. But some one may perhaps be thinking and anxious for the time to arrive when he can say—suppose we grant all that, what use is it. what advantage is there in it, what more do we know of the mineral nutrients and their role in metabolism, what more do we know of dietetics, how does this contention in any way aid the progress of scientific dietetics, what is the practical good of your paper any how?

I would like to conclude with some observations more or less practical in character, and more or less intended to answer some of these inquiries. As to the elemental motive of this paper, I would like to observe that it comes from my conviction that the union of the sciences of botany and zoology in the single science of biology is one of the most energising, illuminating, potential, seminal facts of the century, one which means much to the medical profession, one which that profession does not seem to appreciate adequately, and one which has a special message of hope and progress to the growing science of dietetics and to dieto-therapeutics. There was profound knowledge in the saying of the ancient, "Man is a plant with his roots inside." There is the wisdom of inspiration in the writer of today when he says, "The more we know of plants and animals the more we find they resemble each other." It is my conviction that our way to a better knowledge of dietetics in man runs through a study of the nutrition of plants and this is primarily and chiefly a study of the mineral nutrients. My impression is distinct that the botanists can teach the doctors many a lesson of value, that the knowledge of the metabolism of plants is more accurate, more demonstrative, more scientific than our notions of the metabolism of animals and man.

Again, we all agree that a badly cooked meal or a meal of improper food like tainted meat or impure milk, is distinctly objectionable and offensive, and we are quick to protest with emphasis against one single offense of this character; and at the same time, possibly, in the same house and even in the same case, we sit day after day and week after week in supine complaisance, at least without protest, to some ten or fifteen thousand meals a day each one of which is impure and tainted, the air of the room warmed by the pipeless stove, by the grate without a chimney, or ventilated by the fan in the basement.

There is something quite like latent Eddyism new thought in the minds of many of us which makes us so unobserving or so tolerant to the dangerous, the most pathogenic of foods,—foul air. In comparatively recent times distinct advance has been made in the diagnosis of the diseases of metabolism, and interesting paragraphs are devoted to the discussion of the evil results of the fermentation of the sugars and starches, and of the putrefaction of the albuminate food. I recall with gratitude the comfort which came to me with my notions as to the value of the mineral nutrients, that at least these foods would neither ferment nor putrify. It is possible that you may not be much impressed by these, the negative virtues of the mineral nutrients, but the longer I study the evils of fermentation and putrefaction, of neurasthenia gastro-colonica, the more I am persuaded that foods free from these dangers have a wide range of opportunity for usefulness.

In the foregoing I had in mind diseases in which metabolism is disturbed in a chronic way. There are other diseases of a more acute character in which metabolism, say for starch or albuminates, is still more seriously interrupted for the time being; for instance, in acute nephritis with convulsions, as in cases of that trouble following scarlet fever. I remember well cases of this kind and the comfort it was to me to recall that not only would the diet of the mineral nutrients not ferment and not undergo putrefaction but, moreover, that my patient had acquired, possibly from his plant ancestors, the habit of storing the invaluable elements of nutrition,—nitrogen and starch and sugar and fats against a day of want, and that these deposits of nutritive treasures were now his and that there were enough of them to support him in safety for months,—provided that the usual supply of the more important nutrients be continued. This positive knowledge gives the physician calmness and courage, enabling him to face the anxious and over anxious parents, and the solicitous and meddlesome friends with encouragement and confident assurance.

One of the most vivid recollections of my life is the hopefulness in the treatment of the acute diseases, involving questions of disturbed metabolism coming from a belief in the effectiveness of the mineral nutrients,—air, water and salts,—in fact, the all sufficiency of these nutrients to answer the demands of metabolism for weeks and weeks together.

The diet, and the diseases of the aged present to us a problem of metabolism of singular interest. Our habits of eating and drinking are formed in early life when the demands for growth and the various activities are enormous. These habits are too frequently followed in later years without intelligence or reason because they are habits. Again, old age is the autumn of life,—the time of preparation for the winter of life,—death. In autumn the nutritional activities of spring, of summer, of harvest, are over, the leaves ripen and are ready to fall at the first touch of frost, the sap recedes and all of the nutritional activities of the tree lessen and lessen almost to the point of complete inactivity. It is the same with man. The demands for growth are long since passed and over, the keenness of nervous sensibility is passing, the demands for the expenditures for procreation are lessening and should be over; the organism has the advantage of the accumulations of years and years of stores of proteids, of sugars, of fats; with lessening demands for nutriment, there is in a marked degree lessening capacity for anabolism and for katabolism; and still in spite of all this, the habits of eating and drinking of the twenties, the thirties and the forties, continue to be the habits of the fifties and the sixties; and chiefly by reason of these habits, so few men reach the seventies and the eighties.

The problems of anabolism and katabolism of the aged in sickness and in health are at once the most intricate, most difficult and most important, which may engage the attention of the medical man. The knowledge, actual and potential of the role in metabolism of the mineral nutrients,—air, water and the salts, —is an invaluable aid in the solution of these problems.

Let it be remembered that the mineral nutrients include the salts of calcium and magnesium, and it will be well here to recall, that in angiosclerosis and with greater particularity, in arteriosclerosis, the pathological deposition of these salts in the diseased tissues is characteristic.

We already know that each food has its minimum, its medium and its maximum, and we are slowly learning that the misuse of foods, by under use, or by over use, is an important factor in the production of disease. I have the most positive conviction that a more thorough knowledge of the role of the mineral nutrients, —salts in metabolism,—will throw important light upon the causation, the prevention, and the treatment of rickets, scurvy, anemia, chlorosis, tuberculosis, diabetes, calculus, myositis ossificans, angiosclerosis, or morbid conditions involutional in character.

The introduction of hypodermoclysis and its rapid growth in confidence and popularity is an instance of the potential value of the mineral nutrient salts in therapeutics, also an apt illustration of the fact that in nutrition the food is the thing, and in general the route, by nose and lung, by mouth and stomach, by colon, by the cellular tissue, or by the veins, is non-essential. However, in a given case the route selected may mean success or failure, life or death. Sodium is an important member of the class of mineral salts and its chloride one of its more important combinations, but it is important to keep always in mind that potassium, calcium, magnesium, phosphorus, sulphur and iron are also members of that class, and it would seem to be absurd to conclude that sodium and its one salt the chloride, should monopolise all the therapeutic potentialities of the group.

To my mind the fact that water bearing a certain portion of sodium chloride in solution, has been found of enormous value in therapeutics when given in hypodermoclysis, in enteroclysis or by the endovenous route, should convince us that each and every one of the mineral nutrient salts has similar therapeutic potentiality. Experiment and observation have only to determine the conditions requiring such medication, and the form in which the remedy is to be administered.

I may here remind the reader that the work done with the solutions of sodium chloride has been for the greater part confined to the use of isotonic solutions. So far as I know the value of this remedy in hypertonic and hypotonic solutions is yet to be demonstrated by experiment and observation.

SUMMARY.

The right classification of foods is a matter of great importance to right living and in the prevention and cure of disease.

In the right classification of foods the mineral nutrients,—air, water, and the salts will constitute class one.

This classification is demanded by our growing knowledge of biology and also in the interest of the art of medicine, that we may the more effectively advise our patients.

433 Franklin Street.

